# Agenesis of the infra-hepatic segment of the inferior vena cava associated with recurrent deep venous thrombosis: case report

**DOI:** 10.1590/1677-5449.210006

**Published:** 2021-09-10

**Authors:** Vinicius Tadeu Ramos da Silva Grillo, Pedro Luciano Mellucci, Rodrigo Gibin Jaldin, Matheus Bertanha, Rafael Elias Fares Pimenta, Marcone Lima Sobreira

**Affiliations:** 1 Universidade Estadual “Júlio de Mesquita Filho” – UNESP, Faculdade de Medicina de Botucatu, Hospital das Clínicas, Botucatu, SP, Brasil.

**Keywords:** inferior vena cava, deep venous thrombosis, cardiovascular abnormalities

## Abstract

Agenesis of the inferior vena cava (IVC) has been described in less than 1% of the population; a rare occurrence caused by embryonic abnormalities. Its correlation with deep vein thrombosis (DVT) is certainly underestimated, since this change is hard to detect using ultrasound. The aim of the article is to report the case of a 41-year-old female patient with pain and edema up to the top of the right thigh after plastic surgery. Bilateral venous duplex ultrasound revealed bilateral DVT involving iliac-femoral-popliteal and distal segments. Venous angiotomography was requested because the IVC was not visible on ultrasound, revealing thrombosis of the right lumbar plexus and iliofemoral segment bilaterally and agenesis of the infrahepatic segment of the inferior vena cava, with ectasia and compensatory tortuosity of paravertebral veins and the azygos-hemiazygos system, and bilateral pelvic varices. Systemic and oral anticoagulation were administered, with a satisfactory clinical response.

## INTRODUCTION

The most common cause of agenesis of the inferior vena cava (IVC) during embryogenesis is dysgenesis with a venous system defect, which may be secondary to intrauterine or perinatal thrombosis, without accompanying embryonic abnormalities.^1^ Anomalies of the IVC have been diagnosed incidentally with greater frequency since the advent of imaging exams, since the majority of patients are asymptomatic.^2^^-^5

Deep venous thrombosis (DVT) is caused by a congenital or acquired hypercoagulable state. Absence of the IVC may be a risk factor, since it impairs drainage of blood from the lower limbs and causes a state of venous stasis and hypercoagulability, in which venous return is dependent on a system made up of collaterals.^1^^-^3^,^^6^^-^10 This study was duly evaluated and approved by a Research Ethics Committee (CAAE 45875521.4.00000.5411 and ruling number 4.699.406).

## DESCRIPTION OF THE CASE

A 41-year-old female patient presented at a vascular surgery service with pain of moderate intensity in the right lower limb, associated with accentuated edema around the hip. Six days earlier she had undergone abdominal liposuction, mastopexy, and breast augmentation with implants.

She had no comorbidities and had been taking combined oral contraception with levonorgestrel 0.15 mg and ethinylestradiol 0.03 mg for the preceding 1 year and 6 months, which was withdrawn after diagnosis since it is a risk factor. She had a personal history of DVT in the left lower limb, in the femoral-popliteal segment, at 22 years of age, during postpartum after a natural delivery, when she had been treated with anticoagulation with a vitamin K antagonist (warfarin) for 1 year, having been discharged to outpatients follow-up after treatment.

Clinical assessment found her in good general health, with no changes to vital signs, with discrete abdominal edema and minor ecchymosis, primarily in the pubic area, with small quantities of serous secretions from the surgical wound in the right iliac fossa. Lower limbs were free from cyanosis and pallor, with moderate bilateral edema, more pronounced on the right, and with palpable distal pulses.

The results of laboratory tests requested at admission included: hemoglobin 11.1 g/dL (normal range [NR]: 13.5-17.5 g/dL), hematocrit 32.3% (NR: 41-53%), platelets 136,000 µL (NR: 140,000-440,000 µL), and white blood count 11,700/µL (NR: 4,000-11,000/µL). Homocysteine, urea, creatinine, albumin, transaminases, coagulogram, and electrolytes were all normal. As such, the patient’s laboratory tests revealed mild anemia, thrombocytopenia, and leukocytosis.

**Figure 1:**
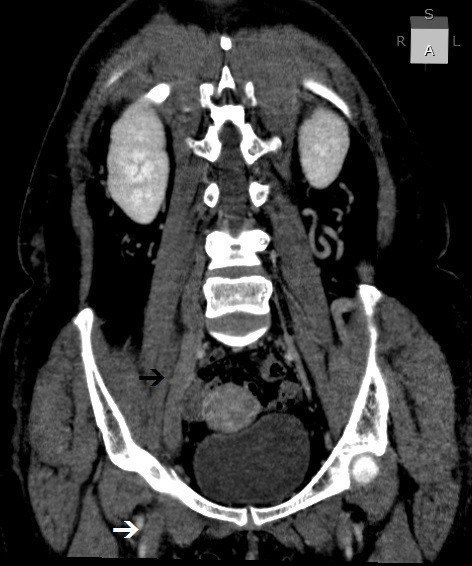
Venous angiotomography, coronal anterior oblique view, showing venous thrombosis of the right common femoral vein (white arrow) and the right lumbar plexus (black arrow).

**Figure 2:**
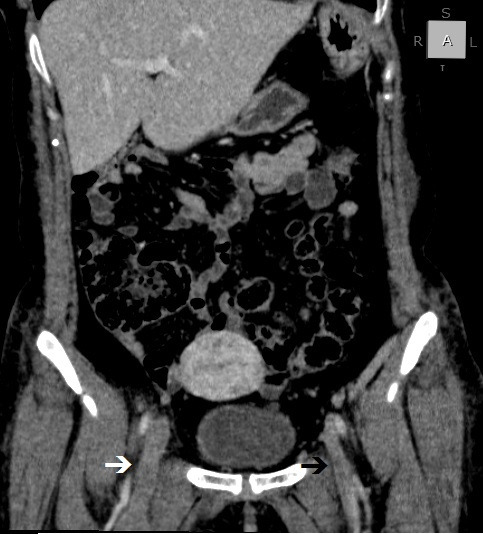
Venous angiotomography, coronal view, showing right (white arrow) and left (black arrow) iliofemoral venous thrombosis.

**Figure 3:**
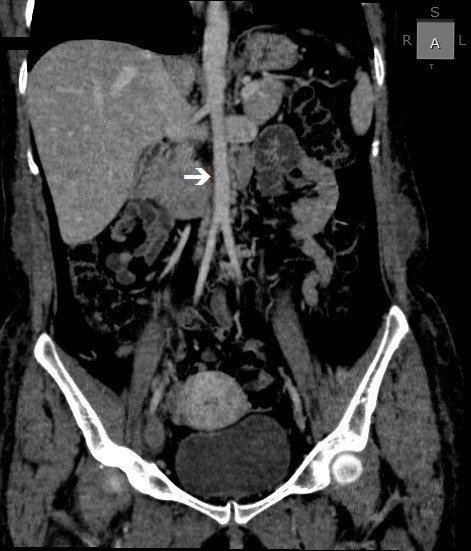
Venous angiotomography, coronal view, revealing agenesis of the infra-hepatic segment of the inferior vena cava. Note the abdominal aorta with no adjacent vascular structure (arrow).

**Figure 4:**
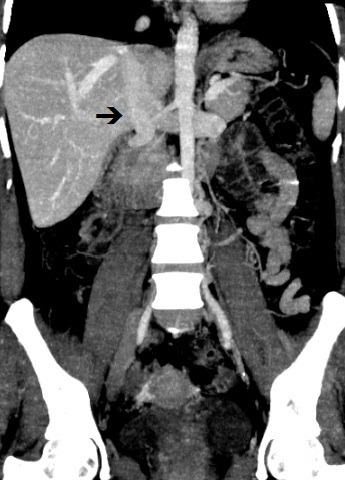
Venous angiotomography, coronal view, showing the origin of the inferior vena cava from the confluence of the renal veins (arrow).

**Figure 5:**
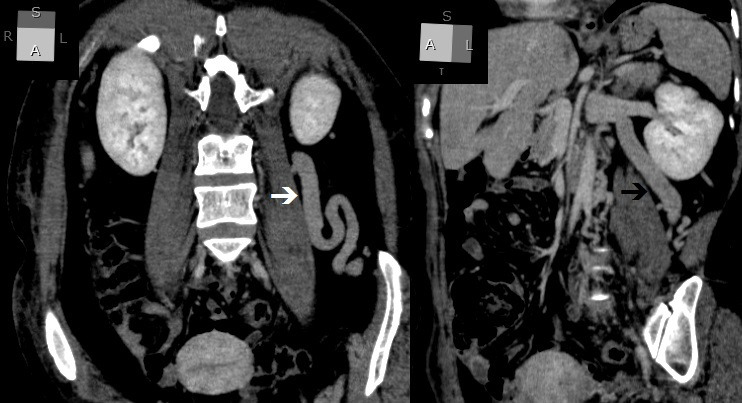
Venous angiotomography, coronal view (left) and coronal oblique view (right), showing tortuosity (white arrow) and ectasia of the left gonadal vein (black arrow).

Vascular ultrasonography with duplex (USD) was used to examine the deep vein system of the lower limbs and the abdominal venous system. These examinations revealed acute DVT in the iliac-femoral-popliteal segment and distal segments bilaterally. The IVC was not visible in the USD examination, and, in view of the patient’s postoperative status with edema of the abdominal subcutaneous tissues, an additional imaging exam was conducted for diagnostic confirmation. Venous angiotomography of the abdomen and pelvis identified venous thrombosis of the right lumbar plexus (Figure 1) and of the iliofemoral segment bilaterally (Figure 2) and agenesis of the infra-hepatic segment of the IVC (Figure 3), which originated at the confluence of the renal veins (Figure 4), with associated compensatory ectasia and tortuosity of the left gonadal vein, paravertebral veins, and the azygos-hemiazygos system (Figure 5), with pelvic varicose veins bilaterally.

The patient was initially treated with systemic anticoagulation in hospital, using unfractionated heparin via a continuous infusion pump at an initial dose of 18 UI/kg/h. The pain and edema in her lower limbs gradually diminished and she was discharged from hospital on the seventh day, with a prescription for indefinite oral anticoagulation with warfarin and instructions to adopt the Trendelenburg position when lying supine, wear mid-thigh medium compression elastic stockings (20-30 mmHg), and attend outpatients follow-up. At periodic outpatients consultations (14, 30, and 60 days and every 90 days thereafter), she was free from complaints, her anticoagulation level was within the proposed target range (prothrombin activity time 2.5-3.5), symptomology had improved, and she had had no bleeding episodes.

## DISCUSSION

Embryological formation of the IVC is a complex process involving many anastomoses of pairs of embryonic veins.^2^^-^4^,^^11^ Development of the infra-hepatic segment of the IVC starts between the sixth and eighth weeks of intrauterine life, and several theories as to its formation have been proposed. Normally, the IVC comprises four segments: the hepatic, derived from the vitelline vein; the suprarenal, which develops from the right subcardinal vein; the renal, from the right supracardinal vein; and the infrarenal, which also derives from the right supracardinal vein.^4^^-^6

Congenital anomalies of the IVC have estimated prevalence de 0.07% to 8.7% in the general population.^2^ Many different anomalies have been described, including an IVC on the left, a double IVC, continuation of the IVC to the azygos, a circum-aortic left renal vein, a retroaortic left renal vein, a circum-caval ureter, agenesis of the hepatic segment of the IVC, and infrarenal agenesis with preservation of the suprarenal segment, as described in the present report.^4^^,^^12^ Agenesis is described in less than 1% of the population; a rare occurrence that is caused by abnormal embryonic development.^7^^,^^13^ The majority of cases of partial absence of the IVC affect its supra-hepatic portion (90%) and there are associations with congenital heart disease in 0.6% to 2% of cases or with other cardiac anomalies in 0.3% to 0.5%.^14^ Agenesis of the infrarenal segment of the IVC is extremely rare, considering that only 6% of these anomalies involve the renal or infrarenal segments.^13^

It has been suggested that agenesis of the IVC should be considered in young patients with proximal DVT that is idiopathic, bilateral, and recurrent, in the absence of predisposing risk factors such as thrombophilia and especially in patients under the age of 30.^1^^-^3^,^^7^^,^^8^^,^^10^^,^^11^^,^^13^^,^^15^^,^^16^ Known risk factors for DVT also have an influence, acting in synergy if combined with IVC agenesis. These include genetic factors that lead to hypercoagulability, such as deficiencies of proteins C and S and antithrombin; factor V Leiden, high concentrations of factor VIII and hyperhomocysteinemia, in addition to acquired risk factors such as traumas, surgery, immobilization, and pregnancy.^9^^,^^10^ Other thrombogenic factors described include intense muscle exercise, long journeys, and oral contraceptives.^2^^,^^3^

The association between IVC agenesis and DVT is undoubtedly underestimated, since this anomaly is unlikely to be detected with USD and other diagnostic methods such as computed tomography or angiography are needed when there is a suspicion of involvement of supra-inguinal segments.^8^^,^^15^^,^^16^

Knowledge of anatomic variations is important to avoid diagnostic errors and so that surgeons are aware of possible intraoperative complications, such as ligation of collateral veins with serious pathophysiologic consequences or even death.^4^^,^^6^^,^^13^^,^^14^ Patients who are diagnosed with these vascular anomalies should be advised to avoid thrombogenic risk factors because of the high risk of thrombosis and recurrence.^2^^,^^10^

To date, there are no reports giving indications for interventional treatments for IVC agenesis and there is insufficient data to recommend prophylactic treatment of these patients. There are also no indications with regard to prolonged use of anticoagulants in patients who have had DVT previously.^10^^,^^11^^,^^13^

## CONCLUSIONS

Diagnostic suspicion is indispensable, particularly in young patients with recurrent proximal DVT, in order to proceed with correct etiologic diagnosis, anticoagulant therapy, and patient guidance. With regard to duration of treatment and the therapeutic target, controlled studies should be conducted to guide conduct in the future.
