# Supplementary material for: Agenesis of the infra-hepatic segment of the inferior vena cava associated with recurrent deep venous thrombosis: case report
Source: J Vasc Bras. 2021 Sep 10;20:e20210006. doi: 10.1590/1677-5449.210006 (PMC9045531; doi:10.1590/1677-5449.210006)
Supplement: Translation

# Translation: Agenesia de segmento infra-hepático de veia cava inferior associada a trombose venosa profunda de repetição: relato de caso

**Authors:** Vinicius Tadeu Ramos da Silva Grillo, Pedro Luciano Mellucci, Rodrigo Gibin Jaldin, Matheus Bertanha, Rafael Elias Fares Pimenta, Marcone Lima Sobreira

**Affiliations:** 1 Universidade Estadual “Júlio de Mesquita Filho” – UNESP, Faculdade de Medicina de Botucatu, Hospital das Clínicas, Botucatu, SP, Brasil.

**Keywords:** veia cava inferior, trombose de veia profunda, anomalias cardiovasculares

## Abstract

A agenesia de veia cava inferior é descrita em menos de 1% da população, de ocorrência rara e devido a alterações embrionárias. Sua correlação com a trombose venosa profunda certamente é subestimada, visto que tal alteração é de difícil detecção pela ultrassonografia. O objetivo deste artigo foi relatar o caso de uma paciente de 41 anos com dor e edema até a raiz de coxa direita após cirurgia plástica. Foi realizado dúplex venoso de membros inferiores e evidenciada trombose venosa profunda ilíaco-femoro-poplíteo e distal bilateralmente. Solicitado angiotomografia venosa devido a não visualização de veia cava inferior no ultrassom, evidenciando trombose de plexo lombar direito e segmento ilíaco-femoral bilateral, além de agenesia de segmento infra-hepático de veia cava inferior, com ectasia e tortuosidade compensatória de veias paravertebrais, sistema ázigos e hemiázigos, com varizes pélvicas bilateralmente. Foi realizada anticoagulação sistêmica e oral, com boa evolução clínica.

## INTRODUÇÃO

A causa mais comum de agenesia da veia cava inferior (VCI) durante a embriogênese é a disgenesia com defeito do sistema venoso, a qual pode ser secundária à trombose intrauterina ou perinatal, sem anormalidades embrionárias associadas^1^. As anomalias da VCI tornaram-se mais diagnosticadas incidentalmente após o advento de exames complementares de imagem, visto que a maioria dos pacientes é assintomática^2^^-^5.

A trombose venosa profunda (TVP) é causada por um estado de hipercoagulabilidade congênita ou adquirida. A ausência da VCI pode ser um fator de risco, visto que altera a drenagem do sangue dos membros inferiores e ocasiona um estado de estase venosa e hipercoagulabilidade, no qual o retorno venoso depende de um sistema venoso formado por colaterais^1^^-^3^,^^6^^-^10. Este estudo foi devidamente avaliado e aprovado por um Comitê de Ética em Pesquisa (CAAE 45875521.4.0000.5411 e parecer número 4.699.406).

## DESCRIÇÃO DO CASO

Paciente com 41 anos, sexo feminino, deu entrada no serviço de cirurgia vascular devido a quadro de dor de moderada intensidade em membro inferior direito, associada a edema rizomélico acentuado. Apresentava-se em sexto dia de pós-operatório de lipoaspiração de abdômen e mastopexia com implante de próteses mamárias.

Não apresentava comorbidades e fazia uso apenas de anticoncepcional oral composto por levonorgestrel 0,15 mg associado com etinilestradiol 0,03 mg durante 1 ano e 6 meses que precederam o quadro, o qual foi suspenso após diagnóstico por apresentar-se como fator de risco. Apresentava antecedente pessoal de TVP em membro inferior esquerdo no segmento femoro-poplíteo aos 22 anos de idade, no puerpério de parto vaginal, sendo tratada à época com terapia anticoagulante com antagonista de vitamina K (varfarina) pelo período de 1 ano, recebendo alta ambulatorial após tratamento.

Durante a avaliação clínica, apresentava-se em bom estado geral, sem alterações dos sinais vitais, com abdômen discretamente edemaciado, além de algumas equimoses, principalmente em região pubiana, com saída de pequena quantidade de secreção serosa pela ferida operatória em fossa ilíaca direita. Membros inferiores sem cianose ou palidez, com edema moderado bilateral, mais pronunciado à direita, com pulsos distais palpáveis.

Entre os exames laboratoriais de entrada, apresentaram-se os seguintes valores: hemoglobina 11,1 g/dL (valores de referência [VR]: 13,5-17,5 g/dL), hematócrito 32,3% (VR: 41-53%), plaquetas 136.000 µL (VR: 140.000-440.000 µL) e leucócitos 11.700/µL (VR: 4.000-11.000/µL). Homocisteína, ureia, creatinina, albumina, transaminases, coagulograma e eletrólitos não mostraram alterações. Portanto, a paciente apresentava, aos exames laboratoriais, anemia, plaquetopenia e leucocitose discretas.

Foi realizado exame de ultrassonografia vascular com Doppler (USD) para o sistema venoso profundo de membros inferiores e sistema venoso abdominal. Nesse exame, foi identificada TVP aguda de segmento ilíaco-femoro-poplíteo e distal bilateralmente. Durante a USD, não foi visualizada a VCI e, devido a quadro de pós-operatório com edema de tecido celular subcutâneo abdominal, optou-se por exame de imagem adicional para confirmação diagnóstica. Foi realizada angiotomografia venosa de abdômen e pelve, que evidenciou trombose venosa de plexo lombar direito (Figura 1) e do segmento ilíaco-femoral bilateral (Figura 2) e agenesia de segmento infra-hepático de VCI (Figura 3), sendo que sua origem estava na confluência das veias renais (Figura 4), associado a ectasia e tortuosidade compensatória de veia gonadal esquerda, veias paravertebrais, sistema ázigos e hemiázigos (Figura 5), com varizes pélvicas bilaterais.

**Figura 1:** Angiotomografia venosa em corte coronal oblíquo anterior, evidenciando trombose venosa da veia femoral comum direita (seta branca) e do plexo lombar direito (seta preta).

**Figura 2:** Angiotomografia venosa em corte coronal, evidenciando trombose venosa ilíaco-femoral direita (seta branca) e esquerda (seta preta).

**Figura 3:** Angiotomografia venosa em corte coronal, evidenciando agenesia de segmento infra-hepático da veia cava inferior. Nota-se a artéria aorta abdominal sem estrutura vascular adjacente (seta).

**Figura 4:** Angiotomografia venosa em corte coronal, evidenciando a origem da veia cava inferior a partir da confluência das veias renais (seta).

**Figura 5:** Angiotomografia venosa em corte coronal (esquerda) e coronal oblíquo (direita), evidenciando tortuosidade (seta branca) e ectasia da veia gonadal esquerda (seta preta).

Como conduta terapêutica, a paciente foi submetida à anticoagulação sistêmica em regime hospitalar, com heparina não fracionada em bomba de infusão contínua na dose inicial de 18 UI/kg/h. Ela evoluiu com redução da dor e edema de membros inferiores e recebeu alta hospitalar no sétimo dia de internação, com prescrição de anticoagulação oral com varfarina por tempo indeterminado, orientações de ficar em posição de Trendelenburg quando em decúbito dorsal, fazer uso de meia elástica de média compressão (20-30 mmHg) sete oitavos e realizar o retorno ambulatorial para seguimento. Nas reavaliações ambulatoriais periódicas subsequentes (14, 30, 60 e a cada 90 dias), a paciente apresentava-se sem queixas, em nível adequado de anticoagulação conforme alvo proposto (tempo de atividade protrombina 2,5-3,5), com melhora da sintomatologia e sem episódios de sangramentos.

## DISCUSSÃO

A formação embriológica da VCI é um processo complexo que envolve diversas anastomoses de pares de veias embrionárias^2^^-^4^,^^11^. Inicia-se o desenvolvimento do segmento infra-hepático da VCI entre a sexta e a oitava semanas da vida intrauterina, com diversas teorias propostas para a sua formação. Normalmente, a VCI compreende quatro segmentos: hepático, derivado da veia vitelina; suprarrenal, que se desenvolve a partir da veia subcardinal direita; renal, a partir da veia supracardinal direita; e infrarrenal, que também deriva da veia supracardinal direita^4^^-^6.

As anomalias congênitas da VCI têm uma prevalência estimada de 0,07% a 8,7% na população em geral^2^. Entre as várias alterações descritas, podemos encontrar VCI à esquerda, sua duplicação, continuação da VCI para ázigos, veia renal esquerda circum-aórtica, veia renal esquerda retroaórtica, ureter circum-caval, agenesia do segmento hepático da VCI e agenesia infrarrenal com preservação do segmento suprarrenal, como descrito neste presente relato^4^^,^^12^. A agenesia é descrita em menos de 1% da população, de ocorrência rara, e decorre de alterações no desenvolvimento embrionário^7^^,^^13^. A maioria dos casos de ausência parcial da VCI afeta a porção supra-hepática (90%), e há associação com cardiopatias congênitas em 0,6% a 2% ou outras anomalias cardíacas em 0,3% a 0,5%^14^. A agenesia do segmento infrarrenal da VCI é extremamente rara, considerando que somente 6% dessas alterações acometem os segmentos renal ou infrarrenal^13^.

Sugere-se que a agenesia de VCI deva ser considerada em pacientes jovens com TVP proximal, idiopática, bilateral, recorrente, na ausência de fatores de risco predisponentes como trombofilia e especialmente em pacientes com menos de 30 anos^1^^-^3^,^^7^^,^^8^^,^^10^^,^^11^^,^^13^^,^^15^^,^^16^. Os fatores de risco conhecidos para TVP também influenciam e atuam de forma sinérgica se associados à agenesia de VCI, como fatores genéticos que levam à hipercoagulabilidade como deficiência de proteínas C, S e antitrombina; fator V de Leiden, altas concentrações de fator VIII e hiperhomocisteinemia, além de fatores de risco adquiridos como traumas, cirurgias, imobilização e gestação^9^^,^^10^. Demais fatores trombogênicos descritos foram exercícios musculares intensos, viagens longas e anticoncepcionais orais^2^^,^^3^.

A associação entre agenesia de VCI e TVP certamente é subestimada, visto que essa alteração é dificilmente detectada à USD, sendo necessários outros métodos diagnósticos quando houver suspeição de envolvimento de segmentos supra-inguinais, tais como: tomografia computadorizada ou angiografia^8^^,^^15^^,^^16^.

O conhecimento das variações anatômicas torna-se importante para evitar erros diagnósticos, além de alertar o cirurgião para possíveis complicações intraoperatórias, como ligaduras de veias colaterais com sérias consequências fisiopatológicas ou até óbito^4^^,^^6^^,^^13^^,^^14^. Pacientes que apresentam o diagnóstico de tais anomalias vasculares devem ser aconselhados a evitar fatores de risco trombogênicos, pelo seu alto risco de trombose e recorrência^2^^,^^10^.

Até o momento, não existem relatos de indicações de tratamentos intervencionistas nas agenesias de VCI, e não há dados suficientes para a orientação de tratamento profilático desses pacientes. Também não há indicações quanto ao uso prolongado de anticoagulantes nos pacientes que apresentaram quadros pregressos de TVP^10^^,^^11^^,^^13^.

## CONCLUSÃO

É imprescindível a suspeição diagnóstica, principalmente nos pacientes jovens com TVP proximal recorrente, para proceder com o correto diagnóstico etiológico, a condução da terapia anticoagulante e a orientação do paciente. Com relação à duração do tratamento e alvo terapêutico, novos estudos controlados deverão ser realizados para nortear a conduta futuramente.
